# Induction of SOCS Expression by EV71 Infection Promotes EV71 Replication

**DOI:** 10.1155/2020/2430640

**Published:** 2020-02-19

**Authors:** Wenying Gao, Min Hou, Xin Liu, Zhaolong Li, Yongjun Yang, Wenyan Zhang

**Affiliations:** The First Hospital of Jilin University, Institute of Virology and AIDS Research & Key Laboratory of Zoonosis, Ministry of Education, College of Veterinary Medicine, Jilin University, Changchun 130021, China

## Abstract

Enterovirus 71 (EV71) is the causative pathogen of hand, foot, and mouth disease (HFMD). However, no effective antiviral therapy is currently available. Some viruses could escape the host's innate immunity by upregulating suppressor of cytokine signaling (SOCS) proteins. Until now, whether EV71 evades the host immune system by regulating the expression of SOCS proteins remains unknown. In this study, we found that EV71 infection promoted SOCS expression at both mRNA and protein levels in vitro and in vivo. Consistently, the infectivity of EV71 was decreased significantly in the SOCS3 or SOCS1 knockdown cells, suggesting that SOCS1 and especially SOCS3 are crucial for EV71 infection. Further investigation showed that SOCS3 promoted virus infection by inhibiting interferon-induced STAT3 phosphorylation. SOCS1 and SOCS3 mRNA expressions were independent on virus-induced type I interferon expression but were blocked by the inhibitor of NF-*κ*B. Therefore, EV71 infection stimulates the expression of SOCS proteins in an interferon-independent way and negatively regulates the JAK/STAT signaling pathway, thus escaping host immunity. All these results may add new information to the mechanism of EV71 in fighting against type I interferon responses.

## 1. Introduction

Enterovirus 71 (EV71), a member of the Picornavirus family, is notable for its role in epidemics of hand, foot, and mouth disease (HFMD) in children, which is a global infectious disease that affects millions of people [1]. Currently, the pathogenic mechanisms underlying EV71 are unclear, and there are no effective treatments for diseases caused by EV71 [2].

Virus infection can induce a large number of antiviral factors and host immune responses [3, 4]. The type I interferon (IFN) (including IFN*α* and IFN*β*) system is a first line of the defense against viral infections [5]. Type I IFN can induce the production of various antiviral factors through the Janus kinase (JAK)/signal transducer and activator of transcription (STAT) signaling pathway [6]. When IFN binds to IFN receptors (IFNAR1 and IFNAR2) on the cell surface, JAK, which is linked to IFNAR1 and IFNAR2, is recruited and activated by phosphorylation. Meanwhile, IFNAR1 is also phosphorylated. STATs form heterodimers after binding to phosphorylated IFNAR1. The activated STATs can enter the nucleus to regulate the transcription of antiviral factors [7].

The suppressor of the cytokine signaling (SOCS) protein family is a classical negative regulator of cytokine receptor signaling of the JAK/STAT pathway and consists of eight structurally similar proteins (SOCS1–7 and cytokine-inducible SH2-containing protein) [8]. SOCS1 and SOCS3 are the most extensively characterized members [9]. Several recent studies have revealed that some viruses could regulate expression of the host proteins by overstimulating SOCS1 and/or SOCS3, thereby inhibiting the immune signaling pathway and facilitating viral evasion of immune surveillance [10, 11]. For instance, HIV infection interferes with the expression SOCS1 and SOCS3, leading to immune activation. Sustained immune activation disrupts the lymphoid system and favors HIV replication [12]. Moreover, hepatitis B virus increases SOCS3 expression, thereby promoting the inflammation in the liver [13]. SOCS1 also aggravates enterovirus-induced cardiac injury [14]. Influenza A virus inhibits type I IFN signaling via NF-*κ*B-dependent induction of SOCS3 expression [15]. However, the relationship of EV71 infection with JAK/STAT/SOCS signaling is less studied. Until now, whether EV71 evades the host immune system by regulating the expression of SOCS proteins remains unknown.

In this study, we investigated the effects of SOCS proteins on EV71 infection and the underlying mechanisms.

## 2. Materials and Methods

### 2.1. Viruses and Cell Lines

EV71 CC063 was isolated from HFMD patients in 2010 [16]. Human embryonic kidney 293T cells (HEK293T, CRL11268, ATCC) and human rhabdomyosarcoma RD cells (CCL-136, ATCC) were maintained in Dulbecco's modified Eagle's medium (DMEM, HyClone) supplemented with 10% fetal bovine serum (FBS, Biological Industries) and penicillin/streptomycin. All cells were cultured at 37°C in a humidified cell incubator with 5% of CO_2_. HEK293T or RD cells were infected with the EV71 virus (CC063) for the different time points.

RD cells were seeded on 24-well culture plates. At 40 to 60% confluence, cells were stimulated with 20 ng/ml IFN*β* for 0 h, 3 h, 6 h, and 9 h, respectively. On the other hand, RD cells were stimulated with 20 ng/ml IFN*β* for 6 h, followed by EV71 infection for 24 h. RD cells were also treated with/without the NF-*κ*B inhibitor (E)-3-(4-methylphenylsulfonyl)-2-propenenitrile (BAY11-7082) for 1 h, followed by EV71 infection, and cells were harvested at 0 h, 3 h, and 9 h after EV71 infection.

### 2.2. Mouse Model of EV71 Infection

The Jilin University Office of Laboratory Animal Management approved our animal care and experimentation procedures, and we carried out the experiments in accordance with accepted guidelines. One-day-old specific-pathogen-free (SPF) ICR neonatal mice (*n* = 36) which were purchased from the Experimental Animal Center (College of Basic Medicine, Jilin University, Changchun, Jilin, China) were used to establish the animal model of viral infection. The neonatal mice were randomly divided into different experimental groups (*n* = 6 each) and inoculated intracerebrally with the EV71 virus CC063 strain (10^3^ CCID50 ml^−1^) or MEM medium (10 *μ*l/mouse), according to previous description [17, 18]. At 0, 2, and 4 days following inoculation, all the mice were sacrificed, and the hind-limb tissue samples of the mice were collected.

### 2.3. Western Blot Analysis

Cells were lysed with lysis buffer (50 mM Tris-HCl, pH 7.8, 150 mM NaCl, 1% NP-40, 1% sodium deoxycholate, and 4 mM EDTA), and then proteins were extracted. Protein concentration was quantified using the BCA assay. Proteins were subjected to SDS-PAGE and then transferred to NC membranes (GE Whatman). The membranes were incubated with primary antibodies and corresponding secondary antibodies. The primary antibodies used in this study are listed as follows: *β*-tubulin monoclonal antibody (Covance), anti-SOCS3 rabbit antibody (Cell Signaling Technology), anti-SOCS1 rabbit antibody (Cell Signaling Technology), anti-STAT3 rabbit antibody (Cell Signaling Technology), and anti-p-STAT3 rabbit antibody (Cell Signaling Technology). The polyclonal antibody against EV71 was obtained from rabbits immunized with EV71 whole viruses in our laboratory. The secondary antibodies were HRP-conjugated anti-rabbit (GE) and anti-mouse (Santa Cruz). The membranes were developed with an ECL substrate (Proteintech). Quantification of protein expression was performed by ImageJ2X software (NIH).

### 2.4. Quantitative Real-Time Polymerase Chain Reaction (RT-PCR)

Total RNA was extracted with a TRIzol reagent (Invitrogen). The cDNA was reverse transcribed with a High-Capacity cDNA Reverse Transcription kit (Roche) and oligo d(T)18 primers. The RT-PCR was carried out on Mx3005P (Agilent Technologies, Stratagene, USA) using the Power SYBR® Green PCR Master Mix (2x) (ABI). The amplification procedure was as follows: initial activation at 95°C for 2 min, followed by 45 cycles of 95°C for 15 s, 57°C for 15 s, and 68°C for 20 s. The primers used in this study are listed in Table 1. Data were normalized to the housekeeping GAPDH gene, and the relative abundance of the transcripts was calculated using Ct methods.

### 2.5. Knockdown of SOCS/IFNAR1 with shRNA

Lentiviruses were produced by transfection of HEK293T cells with pLKO.1-shcontrol or pLKO.1-shSOCS1/3/IFNAR1 together with pRSV-Rev, pMDLg/pRRE, and pCMV-VSVG. The assembled virus-like particles in the culture supernatants were concentrated by ultracentrifugation at 130,000 g for 2 h and then were used to infect fresh HEK293T cells. HEK293T cells were selected with 5 *μ*g/ml puromycin (Sigma) 48 h after infection and then cultured for 1 week in the presence of 5 *μ*g/ml puromycin. SOCS1/3 expression was monitored by RT-PCR and western blot. IFNAR1 expression was monitored by RT-PCR. The shSOCS1, shSOCS3, and shIFNAR1 sequences are as follows: shSOCS1: CCGGCTTCCGCACATTCCGTTCGCACTCGAGTGCGAACGGAATGTGCGGAAGTTTTTG, shSOCS3: CCGGCGGCTTCTACTGGAGCGCAGTCTCGAGACTGCGCTCCAGTAGAAGCCGTTTTTG, and shIFNAR1: CCGGAAGAACTACAGCAGGACTTTGCTCGAGCAAAGTCCTGCTGTAGTTCTTTTTTTG.

### 2.6. Statistical Analysis

All data represent at least three independent experiments and are expressed as the mean ± standard deviation (SD). Statistical comparisons between two groups were made using Student's *t*-test. The statistical significance was defined as follows: NS, no significance; ^∗^*p* < 0.05, ^∗∗^*p* < 0.01, and ^∗∗∗^*p* < 0.001.

## 3. Results

### 3.1. EV71 Virus Infection Induces SOCS1 and SOCO3 Expression

**Figure 1:**
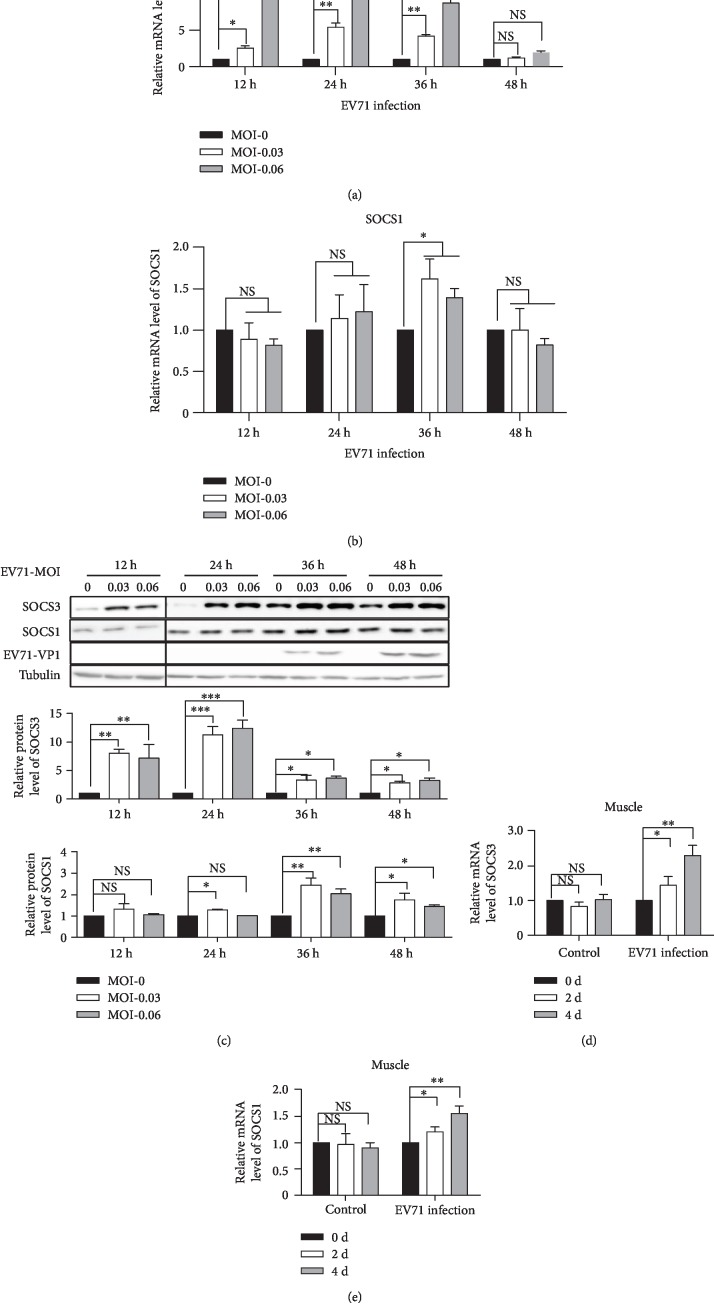
EV71 virus infection induces SOCS expression. (a, b) RD cells were infected with the EV71 virus (CC063) (MOI = 0, MOI = 0.03, and MOI = 0.06) for the indicated time points. The RT-PCR was used to measure SOCS1 (b) and SOCS3 mRNA (a). (c) The expression of the SOCS3/SOCS1 protein was also detected by western blot. Quantification of SOCS3 expression was analyzed by ImageJ2X. SOCS3 expression with EV71 virus infection (MOI = 0) was normalized to 1. (d, e) One-day-old mice were intracerebrally inoculated with EV71 virus at 10^3^ CCID50 ml^−1^ or MEM medium (as control). SOCS1 (e) and SOCS3 (d) mRNA level expressions were assessed by RT-PCR in samples of the hind-limb muscle from the infected mice. Samples were collected at the times indicated. All the data shown in this figure are representative of at least three independent experiments. The statistical significance analyses were performed using two-sided unpaired *t*-test (NS: no significance; ^∗^*p* < 0.05, ^∗∗^*p* < 0.01, and ^∗∗∗^*p* < 0.001).

To examine whether the expressions of SOCS proteins are affected by EV71 infection, RD cells were infected with the EV71 virus and the mRNA levels of SOCS1 and SOCS3 were tested by RT-PCR. The results showed that SOCS1 and SOCS3 mRNA levels increased in the early stages of EV71 infection (Figures 1(a) and 1(b)). In particular, the level of SOCS3 mRNA significantly increased at 12 h, 24 h, and 36 h after infection compared to the negative control (Figure 1(a)). SOCS1 mRNA levels increased at 36 h after infection (Figure 1(b)). In addition, the protein levels of SOCS3 and SOCS1 were analyzed by western blot. The results showed that the expression of SOCS3 significantly increased at 12 h, 24 h, and 36 h after infection (Figure 1(c)), and SOCS1 protein levels increased at 36 h after infection (Figure 1(c)), which was consistent with SOCS mRNA results. In order to further determine whether EV71 infection regulates the expression of SOCS proteins, we infected one-day old mice with the EV71 virus and detected the expression of the SOCS protein in the hind-limb muscle of mice. The results showed that expressions of SOCS1 and SOCS3 were increased significantly in the hind-limb muscle of the EV71-infected mice (Figures 1(d) and 1(e)). Therefore, these results suggest that the levels of SOCS1 and SOCS3 proteins increased during the course of the EV71 infection both *in vitro* and *in vivo*.

### 3.2. SOCS Proteins Promote EV71 Virus Infection

**Figure 2:**
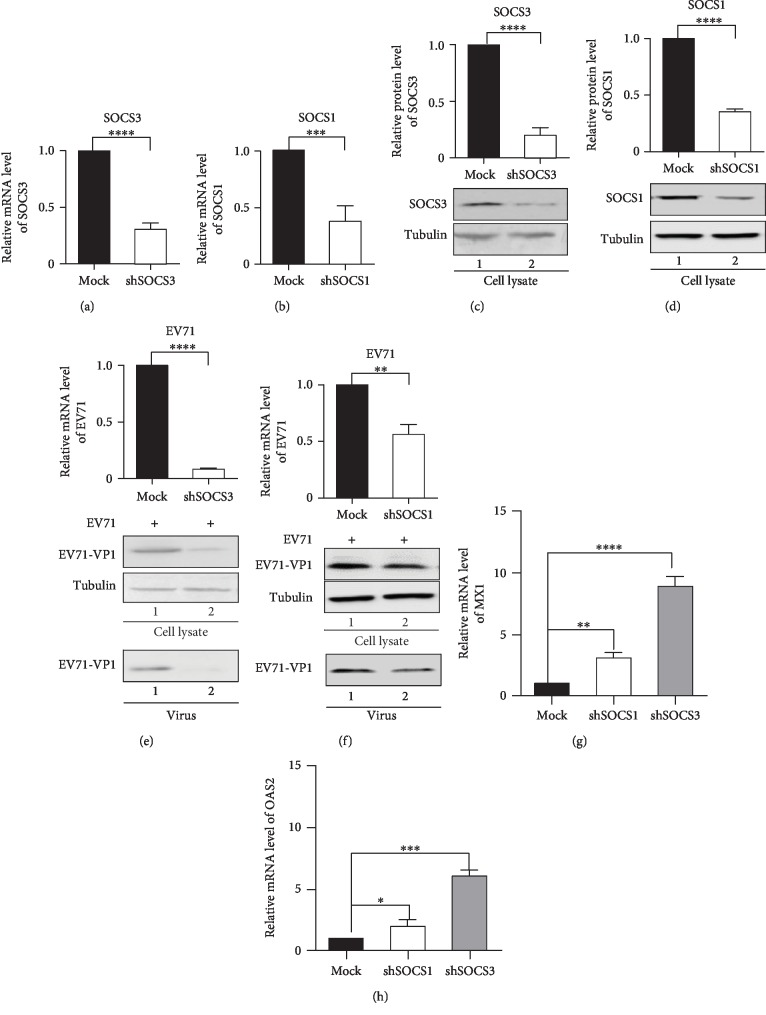
SOCS proteins promote EV71 virus infection. (a–d) Knockdown of SOCS1 and SOCS3 in RD cells was performed with shRNA. The mRNA level expression was determined by RT-PCR (a, b) and the protein level expression was determined by western blot (c, d). (e, f) SOCS knockdown inhibited EV71 virus infection. RD shcontrol cells or stably expressing SOCS1- (f) or SOCS3- (e) specific shRNA cells were stimulated with EV71 (MOI = 0.06). At 36 h after infection, the viral loads were analyzed by RT-PCR (upper) and by western blot (lower). (g, h) Antiviral factor MX1 (g) and OAS2 (h) mRNA expressions were detected in SOCS1 or SOCS3 knockdown RD cells by RT-PCR. The statistical significance analyses were performed using two-sided unpaired *t*-test (^∗^*p* < 0.05, ^∗∗^*p* < 0.01, and ^∗∗∗^*p* < 0.001).

We then investigated the roles of SOCS1 and SOCS3 in EV71 infection. We knocked down SOCS1 and SOCS3 via shRNA in RD cells prior to viral infection. When SOCS3 and SOCS1 were knocked down in RD cells, RT-PCR and western blot results showed significant decreases in mRNA and protein levels of SOCS3 and SOCS1, respectively (Figures 2(a)–2(d)). Furthermore, the production of EV71 in the SOCS3 or SOCS1 knockdown cells was significantly lower than that in the control cells after EV71 infection, which revealed that the infectivity of EV71 was decreased significantly in the SOCS3 or SOCS1 knockdown cells (Figures 2(e) and 2(f)).

Myxovirus resistance 1 (Mx1) [19] and 2′-5′-oligoadenylate synthetases 2 (OAS2) [20] are IFN-induced antiviral genes. SOCS proteins regulate IFN by negative feedback, thereby inhibiting the expression of antiviral factors Mx1 and OAS2 [20, 21]. In order to identify whether there is a similar mechanism involved in EV71 infection, we detected the expression levels of Mx1 and OAS2 in the SOCS3 or SOCS1 knockdown RD cells during EV71 infection. The results showed that the expressions of MX1 and OAS2 were both increased significantly comparing with normal cells (Figures 2(g) and 2(h)). Taken together, these data indicate SOCS1 and especially SOCS3 expression are required for EV71 replication.

### 3.3. Induction of SOCS Is in an IFN-Independent Manner in Early Stage of EV71 Infection

**Figure 3:**
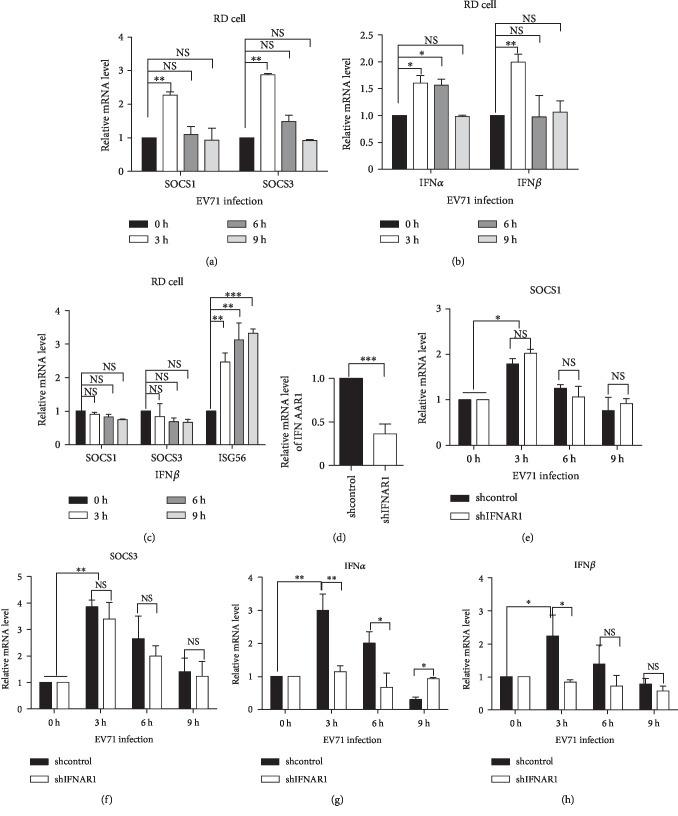
Early induction of SOCS gene transcription is an IFN-independent manner. (a–c) RD cells were infected with EV71 (MOI = 1.5) or stimulated with human IFN*β* for the indicated time points. The mRNA levels of SOCS1 and SOCS3 (a, c), IFN*α* and IFN*β* (b), and ISG56 (c) were analyzed by RT-PCR. (d) Knockdown of IFNAR1 in HEK293T cells was performed with shRNA and determined by RT-PCR. (e–h) The effect of EV71 on SOCS1/3 expression in IFNAR1 knockdown cells. The mRNA levels of SOCS1 and SOCS3 (e, f) and IFN*α* and IFN*β* (g, h) were analyzed by RT-PCR. All the data were representative of at least three independent repeats. The statistical significance analyses were performed using two-tailed unpaired *t*-test (NS: no significance; ^∗^*p* < 0.05, ^∗∗^*p* < 0.01, and ^∗∗∗^*p* < 0.001).

To detect the relationship between EV71 infection and SOCS expression, the EV71 viral titer was increased to MOI 1.5 in the infection of RD cells. SOCS1 and SOCS3 mRNA levels were increased in the early stage of viral infection (Figure 3(a)), accompanied with increased mRNA levels of IFN*α* and IFN*β* (Figure 3(b)). We wonder whether SOCS3 induction might be due to the EV71 virus itself or the paracrine stimulation by IFN during the EV71 infection. Therefore, RD cells were stimulated with IFN*β* for different time points, and the mRNA levels of SOCS1, SOCS3, and the IFN-stimulated gene 56 (ISG56) were measured by RT-PCR. ISG56 is a typical IFN-inducible gene [22], serving as a control. The results in Figure 3(c) showed that ISG56 mRNA was significantly upregulated, whereas neither SOCS1 nor SOCS3 mRNA was significantly elevated in IFN*β*-stimulated RD cells.

To further confirm these results, we knocked down the IFNAR1 in HEK293T cells. RT-PCR showed that about 60% of IFNAR1 was knocked down (Figure 3(d)). The expressions of SOCS1/3 and IFN*α*/*β* were increased in control cells infected with EV71 (Figures 3(e)–3(h)). In the IFNAR1 knockdown cells infected with EV71, the induction of type I IFN was inhibited significantly (Figures 3(g) and 3(h)), while the expressions of SOCS1/3 were still increased (Figures 3(e) and 3(f)). SOCS1/3 levels in the knockdown cells were almost unchanged compared to control cells (Figures 3(e) and 3(f)). These results indicate that induction of the SOCS1 and SOCS3 mRNA expressions is independent on the virus-induced type I IFN expression. SOCS expression may be induced by other signaling pathways during EV71 infection.

### 3.4. EV71 Infection Activates SOCS Expression through NF-*κ*B Pathway and Inhibits IFN-Induced STAT3 Phosphorylation

**Figure 4:**
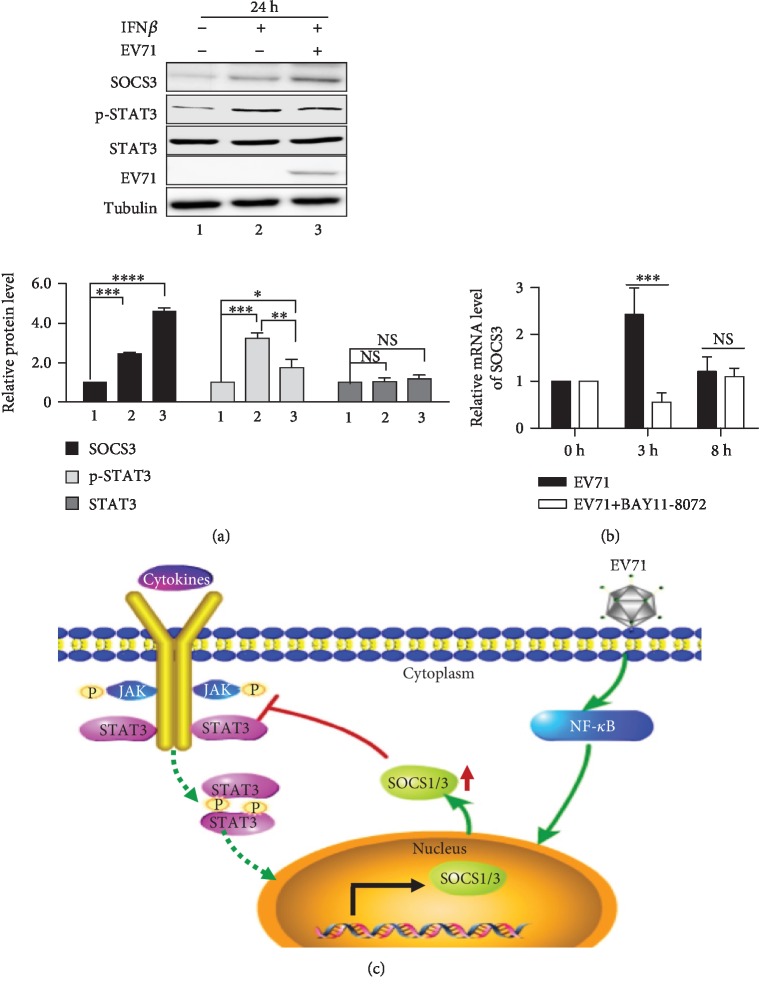
EV71 infection activates SOCS expression through the NF-*κ*B pathway and inhibits IFN-induced STAT3 phosphorylation. (a) RD cells were stimulated with or without IFN*β* for 6 h and then infected with or without EV71 virus for 24 h. The expression of SOCS3, as well as STAT3 phosphorylation, was analyzed by western blot. The relative expressions of SOCS3, p-STAT3, and STAT3 were normalized to those of lane 1. (b) RD cells were treated with NF-*κ*B inhibitor BAY11-7082 for 1 h and then infected with EV71 virus at the indicated time points. SOCS3 expression was detected by RT-PCR. The data are representative of at least three independent repeats. The statistical significance analyses were performed using two-tailed unpaired *t*-test (NS: no significance; ^∗^*p* < 0.05, ^∗∗^*p* < 0.01, and ^∗∗∗^*p* < 0.001). (c) Schematic diagram of the EV71 virus activating SOCS gene expression and inhibiting the JAK/STAT signaling pathway.

We have identified that the expression levels of type I IFN mRNA were induced in EV71 infection cells. However, the relationship between EV71 infection and the JAK/STAT signaling pathway remains unclear. To detect the effects of EV71 infection on the JAK/STAT signaling pathway, we analyzed the expression of related factors by western blot. RD cells were stimulated with 20 ng/ml IFN*β* for 6 h followed by EV71 infection for 24 h. As shown in Figure 4(a), without IFN*β* stimulation and EV71 infection, the expression level of SOCS3 and the phosphorylation level of STAT3 were extremely low. With IFN*β* stimulation and without EV71 infection, the expression level of SOCS3 and the phosphorylation level of STAT3 were significantly increased. However, in the IFN*β*-treated cells infected with EV71, the expression of SOCS3 was further upregulated significantly. Meanwhile, the phosphorylation level of STAT3 was significantly decreased in cells with IFN*β* treatment and EV71 infection than in those with IFN*β* treatment and without EV71 infection, although still significantly higher than those without IFN*β* stimulation and EV71 infection. These data demonstrate that IFN*β* could activate STAT3 phosphorylation, but EV71 infection could inhibit STAT3 phosphorylation by inducing SOCS3 expression.

Furthermore, to explore how EV71 infection induces high expression of SOCS1 and SOCS3, RD cells were treated with/without NF-*κ*B inhibitor Bay11-7082 for 1 h, followed by EV71 infection, and SOCS3 expression was detected by RT-PCR. It was observed that there was a significant reduction in the expression of SOCS3 mRNA in the early stage of viral infection treated with the NF-*κ*B inhibitor compared with the noninhibitor-treated EV71-infected cells (Figure 4(b)). The above results indicate that EV71 infection could activate the SOCS1 and SOCS3 expressions through the NF-*κ*B signaling pathway and counteract the host immune responses in particular by targeting the JAK/STAT signaling pathway, which may be one of the strategies for the EV71 virus to evade host immunity (Figure 4(c)).

## 4. Discussion

Type I IFN response is the most powerful innate immune defense of the body, which can limit the replication and spread of many viruses. Like other viruses, EV71 can not only induce type I IFN response, but also antagonizes its effects through various ways to achieve the purpose of immune escape. SOCS proteins provide selectively negative feedback to prevent overstimulation of the immune system. Because of the immunomodulatory effects of SOCS proteins, it is not surprising that the many viruses hijack SOCS proteins to escape the host immune responses, such as herpes simplex virus 1 (HSV-1) and hepatitis C virus. It was reported that SOCS3 was induced at the very early stage of HSV-1 infection in human amnion cells FL and was accompanied by JAK phosphorylation within 1 h after infection [23]. Further study has shown that in HSV-1-infected FL cells, JAK3 signaling induced SOCS3 expression and then negatively regulated the antiviral IFN*α*/*β* signal, thereby promoting viral replication [23]. However, whether SOCS proteins are hijacked by the EV71 virus to escape host immunity remains unclear.

In this study, we demonstrated that the EV71 virus infection induced SOCS1 and SOCS3 mRNA level expressions both *in vitro* and *in vivo*, and SOCS proteins promoted EV71 virus replication. The SOCS3 is well known as a feedback inhibitor of the JAK/STAT3 signaling pathway [22]. JAK/STAT signaling pathway can activate type I IFN [24]. The expression of antiviral factors OAS2 and MX1 depends on type I IFN regulation [20, 21]. We found that, in the SOCS3 or SOCS1 knockdown RD cells, the expressions of antiviral factors MX1 and OAS2 were significantly increased compared with those of normal cells. We speculate that EV71 might induce SOCS1 and SOCS3 expressions to regulate antiviral factors through the JAK/STAT pathway and then promote viral replication.

Various viruses suppress IFN-driven immunity by inducing SOCS1 and SOCS3, thereby evading immune responses [2]. SOCS proteins can be quickly induced by IFN signaling and inhibit the specific JAK/STAT signaling pathway [25, 26]. This prompted us to investigate whether SOCS1/3 transcription might be induced due to the EV71 virus itself or paracrine action of IFN during EV71 virus infection. In our study, SOCS1 and SOCS3 were increased in the early stage of viral infection in RD cells infected with an increased titer of EV71, accompanied by increased IFN*α* and IFN*β*. These results were further identified by knockdown of IFNAR in HEK293T cells by shRNA approach, in which the induction of type I IFN was inhibited, but the induction of the SOCS1/3 expression was not impaired when cells were infected with EV71. Our results indicate that the SOCS expression might be induced by other signaling pathways in the early stage of EV71 virus viral infection.

The critical role of SOCS3 is manifested by its binding to both JAKs and cytokine receptors, which results in the inhibition of STAT3 phosphorylation. STAT3 triggers a variety of gene expressions in response to cytokine (such as IL-6 and IL-10) and growth factor stimulation and plays a critical role in many cellular biological processes involved in anti-/proinflammatory responses, cell growth, and cell death [27, 28]. However, we demonstrated that early EV71 infection quickly activated SOCS protein expression and inhibited IFN-induced STAT3 phosphorylation. The EV71 virus may escape host immunity by inducing expressions of SOCS1 and SOCS3 to inhibit STAT3 phosphorylation. Meanwhile, we found that the NF-*κ*B inhibitor could inhibit the expression of the SOCS protein induced by EV71. Consistently, Collins et al. reported that murine hepatitis C virus could induce the expression of SOCS3 through the NF-*κ*B pathway, ultimately leading to an increase of the viral titer [29]. Similar results have also been found in the Japanese encephalitis virus [26]. Although EV71 infection initiates a very early type I IFN response, it also activates SOCS protein expression via the NF-*κ*B signaling pathway to block this antiviral strategy.

## 5. Conclusions

In summary, our study found that EV71 virus infection induced SOCS mRNA level expression *in vitro* and *in vivo*. SOCS1 and SOCS3 may promote virus infection by inhibiting IFN-induced STAT3 phosphorylation. In the early stage, the induction of SOCS gene transcription was an IFN-independent manner and could be blocked by the NF-*κ*B inhibitor. All these results may add a new aspect to our knowledge of the strategies used by the EV71 virus to fight against type I IFN responses.

## Figures and Tables

**Table 1:** Primers used in this study.

Primer	Sequence (5′-3′)
SOCS1-RT-F	ACCAGGTGGCAGCCG
SOCS1-RT-R	GTGCGGAAGTGCGTGTC
SOCS3-RT-F	GGCACCTTTCTGATCCGCGACAGCTC
SOCS3-RT-R	GGGCGAGAAGATCCCCCTGGTGTTG
EV71-RT-F	CAAGGGATGGTACTGGAAGT
EV71-RT-R	GATCGGTAGAGGTAGTGGAA
MX1-RT-F	AGATAAGTGGAGAGGCAAGG
MX1-RT-R	CTCCAGGGTGATTAGCTCA
OSA2-RT-F	AGTCTTAAGAGGCAACTCCG
OSA2-RT-R	AAGGGACTTCTGGATCTCG
ISG56-RT-F	TCGGAGAAAGGCATTAGATC
ISG56-RT-R	GACCTTGTCTCACAGAGTTC
IFN*α*-F	TTGGCTGTGAAGAAATACTTCC
IFN*α*-R	GTTTGTTGATAAAGAGAGGGAT
IFN*β*-RT-F	AAACTCATGAGCAGTCTGCA
IFN*β*-RT-R	AGGAGATCTTCAGTTTCGGAGG
IFNAR1-RT-F	TCCAGAAGTACATTTAGAAGC
IFNAR1-RT-R	CTACACCTGAAGAGTTTTTCC
GAPDH-F	GCAAATTCCATGGCACCGT
GAPDH-R	TCGCCCCACTTGATTTTGG

## Data Availability

The data used to support the findings of this study are available from the corresponding author upon request.
